# Supplementary material for: Differential Ability of Spike Protein of SARS-CoV-2 Variants to Downregulate ACE2
Source: Int J Mol Sci. 2024 Jan 22;25(2):1353. doi: 10.3390/ijms25021353 (PMC10816870; doi:10.3390/ijms25021353)
Supplement: Supplementary file 1 [file ijms-25-01353-s001.zip › ijms-2796756-supplementary.pdf]

Supplementary Figures

Fig. S1

A

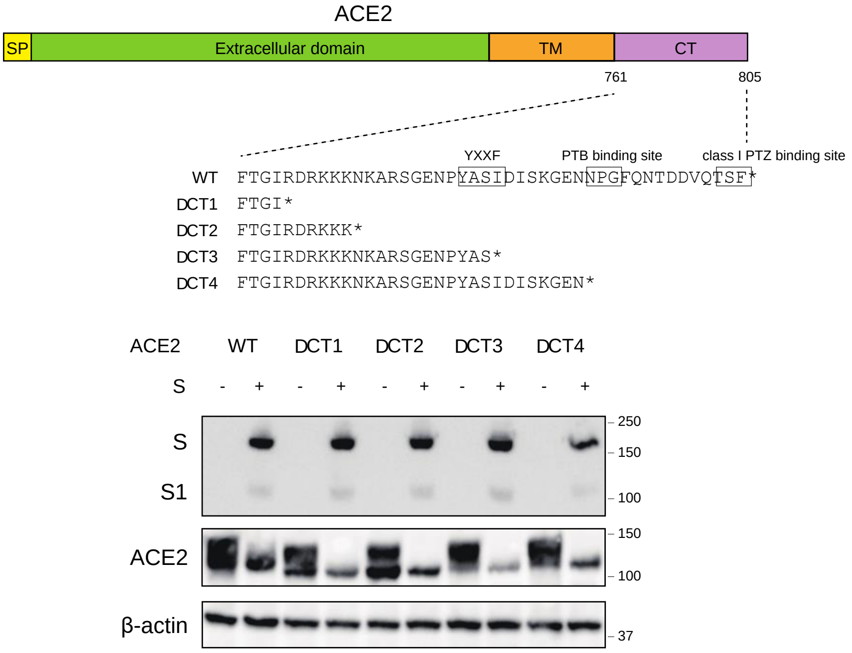

B

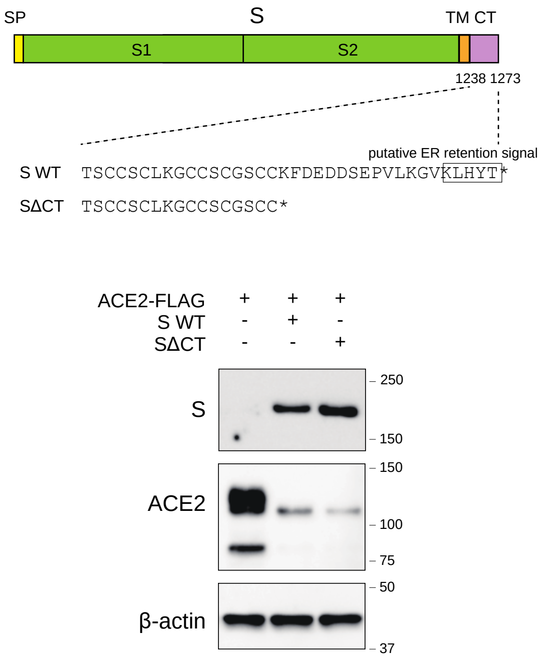

Supplemental Figure S1: Effect of cytoplasmic tail deletions of ACE2 or the S protein of SARS-COV-2 on the downregulation of ACE2. (**A**) (**upper**) Schematic of deletions in

cytoplasmic tail of ACE2. Amino acid sequences of wild-type cytoplasmic tail (WT) of ACE2 or deletions of the cytoplasmic tail (ΔCT1-4) are shown. (**lower**) Cell lysates from 293T cells transfected with ACE2 expression vectors with wild-type (WT) or cytoplasmic deletions (ΔCT1-4) in the presence or absence of an S protein expression vector were subjected to Western blotting using anti-S1, anti-ACE2, and anti-β-actin antibodies. (B) (**upper**) Schematic of a deletion in cytoplasmic tail of the S protein. Amino acid sequences of a wild-type cytoplasmic tail (S WT) or a cytoplasmic tail deletion (SΔCT) of the S protein are shown. (**lower**) Cell lysates from 293T cells transfected with an ACE2-FLAG expression vector in the presence of empty, S WT, or SΔCT vectors were subjected to Western blotting using anti-S1, anti-FLAG, and anti-β-actin antibodies. The positions of the molecular mass marker (kDa) are indicated on the right.
